# The challenge of weight gain in hormone receptor-positive breast cancer

**DOI:** 10.18632/oncoscience.608

**Published:** 2024-09-23

**Authors:** Terrence C. Tsou, Avonne Connor, Jennifer Y. Sheng

**Affiliations:** ^1^Department of Oncology, Johns Hopkins University School of Medicine, Baltimore, MD 21205, USA; ^2^Department of Epidemiology, Johns Hopkins Bloomberg School of Public Health, Baltimore, MD 21205, USA

**Keywords:** breast cancer, weight gain, obesity, health disparities, hormone receptor-positive

Obesity is associated with an increased risk of developing breast cancer [1], and compared to those without, women with obesity are two times more likely to die from breast cancer [2]. There are also persistent health disparities in breast cancer and obesity trends. Among those with early stage breast cancer, Black women experience higher rates of obesity and obesity-related comorbidities (hypertension, heart disease, diabetes) compared to white women [3], and black women have 40% higher and over double the breast cancer mortality compared to their white and Asian/Pacific Islander counterparts, respectively [4].

This study [5] focused on characterizing changes in BMI (body mass index) over time among early-stage breast cancer survivors with hormone receptor (HR) - positive breast cancer. A secondary goal was to determine if an interaction between race and BMI existed and would predict recurrence. Using a single tertiary institution’s database of all patients with breast cancer diagnosed between 2010–2020, our group identified 140 (12.9% Black) patients with early-stage invasive breast cancer who were of female sex with HER-2 negative and ER- and/or PR-positive cancer. A medical record abstraction collected demographic information, BMI within 3 months of diagnosis, most recent BMI at time of chart review, clinical parameters (cancer characteristics, pathology, and treatment), and recurrence. We found that a significantly higher proportion of Black patients had obesity (BMI ≥30 kg/m^2^) at diagnosis compared to other races (55.6% vs. 30.3%, *p* = 0.034). Compared to those without, patients with obesity at diagnosis were also more likely to receive a lumpectomy. Interestingly, a larger proportion of patients with obesity at baseline had a BMI decrease of 0.6 points compared to other participants. Of those with obesity at baseline, only 31% had a BMI increase of at least 0.1/year, compared to 71.4% of those with overweight and 45.5% of those with a healthy BMI. A logistic regression model accounting for high cancer stage (stage >1), Nottingham grade (grade >1), and cancer size (“T” of TNM staging >1) found Black patients, compared to other races, were more likely to have a recurrence (OR 7.21; 95% CI 1.48, 35.09) but the interaction between race and obesity was not statistically significant (*p* = 0.998).

This study highlighted patterns of weight gain among early-stage HR+ breast cancer, especially among those with healthy BMI or overweight after cancer diagnosis. This finding matched a longitudinal epidemiological study [6] in the general population and identified that these two groups would most likely benefit from weight reduction interventions, as those with obesity were more likely to lose weight– possibly due to unintentional weight loss associated with malignancy. A new study also supported this result and found healthy BMI at diagnosis (though not overweight) to be an independent risk factor for 5% weight gain in early stage breast cancer survivors [7]. In addition, a secondary analysis of a prospective cohort study confirmed the relevance of weight gain in HR+ cancer by reporting the 5-year cumulative incidence of clinically significant weight gain (increase of ≥5% of baseline) after adjuvant endocrine therapy reached 52% in a group of women with HR-positive stage 0–III breast cancer [8]. Perhaps more importantly, worsening patient-reported outcomes (PRO) scores that met or exceeded the minimal important difference of the PRO measure at 3 and/or 6-months post-treatment initiation were common and associated with clinically significant weight gain during 5 years of adjuvant endocrine therapy among both pre- and post-menopausal women [8]. As PROs are captured more regularly in breast cancer survivors, it may bolster the importance of weight surveillance and obesity treatment, in order to reduce symptom burden.

As we assess the impact of excess adiposity on the outcomes of patients with HR+ breast cancer, topics regarding obesity treatment and definitions of obesity warrant further consideration. First, the landscape of anti-obesity drugs ushers in new options for weight loss. While glucagon-like peptide receptor agonists have surged in popularity as a weight loss drug, the safety of these drugs and other anti-obesity drugs requires further monitoring among patients with breast cancer and breast cancer survivors [9]. In addition, new guidelines have expanded metabolic and bariatric surgery to all patients with BMI >35 kg/m^2^ regardless of comorbidity burden, providing survivors new tools for weight loss after breast cancer treatment [10]. Weight loss strategies also should be tailored to the needs of the increasing number of young adults developing breast cancer, especially since young women and those with premenopausal status at the time of diagnosis are more likely to gain weight [11]. Furthermore, weight loss and adiposity have mostly been measured through BMI, but other measures of body composition more accurately [12] capture body adiposity and muscle mass distribution with better prognostic value in breast cancer. Ultimately, the development and implementation of effective and scalable weight loss approaches may help to bridge the gaps in health equity among Black and underrepresented women with obesity and breast cancer.

## References

[1] Neuhouser ML, et al. JAMA Oncol. 2015; 1:611–21. 10.1001/jamaoncol.2015.1546. 26182172 PMC5070941

[2] Picon-Ruiz M, et al. CA Cancer J Clin. 2017; 67:378–97. 10.3322/caac.21405. 28763097 PMC5591063

[3] Nyrop KA, et al. Cancer. 2021; 127:922–30. 10.1002/cncr.33288. 33284988

[4] Giaquinto AN, et al. CA Cancer J Clin. 2022; 72:524–41. 10.3322/caac.21754. 36190501

[5] Goyal A, et al. Curr Oncol. 2022; 29:4090–103. 10.3390/curroncol29060326. 35735435 PMC9222132

[6] Stenholm S, et al. Epidemiology. 2015; 26:165–68. 10.1097/EDE.0000000000000228. 25643097 PMC4323564

[7] Sedjo RL, et al. J Cancer Surviv. 2014; 8:410–18. 10.1007/s11764-014-0351-9. 24599421 PMC4127359

[8] Uhelski AR, et al. J Cancer Surviv. 2023. [Epub ahead of print]. 10.1007/s11764-023-01408-y. 37261654 PMC11424737

[9] Cuttica CM, et al. Nutrients. 2023; 15:3737. 10.3390/nu15173737. 37686769 PMC10490004

[10] Eisenberg D, et al. Obes Surg. 2023; 33:3–14. 10.1007/s11695-022-06332-1. 36336720 PMC9834364

[11] Qin B, et al. J Clin Oncol. 2022; 40:2213–23. 10.1200/JCO.21.02973. 35333586 PMC9273374

[12] Bates DDB, et al. AJR Am J Roentgenol. 2022; 219:671–80. 10.2214/AJR.22.27749. 35642760

